# Limitations of Early Serum Creatinine Variations for the Assessment of Kidney Injury in Neonates and Infants with Cardiac Surgery

**DOI:** 10.1371/journal.pone.0079308

**Published:** 2013-11-11

**Authors:** Mirela Bojan, Vanessa Lopez-Lopez, Philippe Pouard, Bruno Falissard, Didier Journois

**Affiliations:** 1 Department of Anesthesia and Critical Care, Necker - Enfants Malades University Hospital, Assistance Publique - Hôpitaux de Paris, Paris, France; 2 Inserm, U669, Paris Sud University and Paris Descartes University, Paris, France; 3 Paul Brousse Hospital, Assistance Publique - Hôpitaux de Paris, Paris, France; 4 Paris Descartes University, Paris, France; 5 Department of Anesthesia and Critical Care, Georges Pompidou European University Hospital, Assistance Publique - Hôpitaux de Paris, Paris, France; Center for Molecular Biotechnology, Italy

## Abstract

**Background:**

Changes in kidney function, as assessed by early and even small variations in serum creatinine (ΔsCr), affect survival in adults following cardiac surgery but such associations have not been reported in infants. This raises the question of the adequate assessment of kidney function by early ΔsCr in infants undergoing cardiac surgery.

**Methodology:**

The ability of ΔsCr within 2 days of surgery to assess the severity of kidney injury, accounted for by the risk of 30-day mortality, was explored retrospectively in 1019 consecutive neonates and infants. Patients aged ≤ 10 days were analyzed separately because of the physiological improvement in glomerular filtration early after birth. The Kml algorithm, an implementation of k-means for longitudinal data, was used to describe creatinine kinetics, and the receiver operating characteristic and the reclassification methodology to assess discrimination and the predictive ability of the risk of death.

**Results:**

Three clusters of ΔsCr were identified: in 50% of all patients creatinine decreased, in 41.4% it increased slightly, and in 8.6% it rose abruptly. Mortality rates were not significantly different between the first and second clusters, 1.6% [0.0–4.1] vs 5.9% [1.9–10.9], respectively, in patients aged ≤ 10 days, and 1.6% [0.5–3.0] vs 3.8% [1.9–6.0] in older ones. Mortality rates were significantly higher when creatinine rose abruptly, 30.3% [15.1–46.2] in patients aged ≤ 10 days, and 15.1% [5.9–25.5] in older ones. However, only 41.3% of all patients who died had an abrupt increase in creatinine. ΔsCr improved prediction in survivors, but not in patients who died, and did not improve discrimination over a clinical mortality model.

**Conclusions:**

The present results suggest that a postoperative decrease in creatinine represents the normal course in neonates and infants with cardiac surgery, and that early creatinine variations lack sensitivity for the assessment of the severity of kidney injury.

## Introduction

The glomerular filtration rate (GFR) has traditionally been used to characterize kidney function and injury, and serum creatinine (sCr) is a widely-used clinical tool to assess GFR. The pathogenesis of acute kidney injury (AKI) following cardiac surgery with cardiopulmonary bypass (CPB) is complex: oxidative stress due to the generation of free hemoglobin and iron through hemolysis; impairment of oxygen delivery to an already hypoxic renal medulla due to hemodilution; alterations in systemic inflammatory mediators caused by regional ischemia; lack of pulsatility of the renal blood flow on bypass; and embolic events during surgery [1]. It is largely assumed that the initial pathologic lesion is acute tubular necrosis [1], and that a reduction in GFR occurs subsequently in severely injured kidneys [2]. Thus, the earliest sign of AKI may not be a decline in GFR. Moreover, an increase in sCr implies the loss of about 50% of the glomerular function. These raise the question of the adequate assessment of the postoperative kidney function by sCr.

Despite all of the shortcomings of sCr as an assessor of AKI [3], current diagnostic and staging criteria are based on abrupt variations in sCr (ΔsCr) [4]–[6]. An increasing severity of AKI is a commonly accepted risk factor of increased postoperative mortality [7]–[10], and even small increases in sCr have been shown to affect survival in adults with cardiac surgery [10]. However, several reports have demonstrated a non-linear relationship between early ΔsCr and mortality after cardiac surgery [8], [9], [11], suggesting inaccurate assessment of the kidney function. In children, severity of postoperative AKI has mainly been assessed by the requirement for renal replacement therapy (RRT) [12]–[15]. However, the initiation of RRT remains somewhat subjective as a hard endpoint, depending on urinary output and on physicians practices, and it has been suggested assess AKI severity by hard outcomes, such as the risk of postoperative death [16]. Few studies refer to AKI as assessed by an increase in postoperative sCr in children [17]–[21]. They have reported associations with the length of mechanical ventilation and postoperative stay, but, unlike in adults, only large increases in sCr have been shown to affect survival [17], [19]. The present study aims to describe changes in sCr in neonates and infants undergoing cardiac surgery with CPB, and to explore the ability of early postoperative ΔsCr to assess the severity of kidney injury. To do so, 30-day mortality has been chosen as a hard outcome.

## Materials and Methods

The project was reviewed and approved by the Ethics Committee of the French Society of Thoracic and Cardiovascular Surgery, Paris, France, who waived the need for specific patient or parental consent with regards to the use of records for this research, following the de-identification of patients information.

This retrospective cohort study used data from the files of all neonates and infants who underwent cardiac surgery with CPB from January 1, 2007 through June 30, 2010 at the Necker - Enfants Malades University Hospital, Paris, France. None of the patients were premature neonates at the time of surgery. Patients underwent surgery in normothermia or mild hypothermia with non-pulsatile CPB and warm blood cardioplegia. Reconstruction of the aortic arch was performed with deep hypothermic circulatory arrest (DHCA) and selective cerebral perfusion. Blood product transfusions were guided by the intraoperative hematocrit, which was maintained above 30% during CPB, and increased to 40–45% by the end of CPB. About 1.5 – 2 ml kg^−1^ per min of CPB via conventional ultrafiltration was performed in all patients to increase their hematocrit and reduce extravascular fluid retention. Surgical complexity was accounted for by the Basic Aristotle Complexity score [22]. The only RRT method used was peritoneal dialysis. Indications for dialysis included the following: evidence of fluid overload and positive fluid balance, inadequate urine output (<1 mL kg^−1^ h^−1^) or anuria unresponsive to fluid challenge and intravenous furosemide for at least 4 hours, concomitant low cardiac output syndrome, and acid-base or electrolyte disturbances (pH <7.30, serum K^+^ > 5.5 mmol L^−1^).

The latest sCr measured before surgery was considered the baseline, and the daily postoperative ΔsCr relative to that baseline was calculated in each patient within 7 days of surgery. The sCr was measured by the Jaffé technique, using a Hitachi 917 auto-analyzer (Roche, Meylan, France), calibrated for pediatric levels, and by the enzymatic method adapted on the Hitachi 917 in case of a very low sCr concentration (≤ 20μmol L^−1^). The design of the present study did not allow assessment of urine output and fluid balance.

### Statistical analysis

Missing data were imputed by the expected value from a regression model including correlated variables as predictors. Missing preoperative sCr values were imputed using age as a predictor, and missing CPB variables using age and the surgical complexity score as predictors. Preoperative sCr levels measured within 2 days of birth were considered unreliable [23], treated as missing and imputed.

The rapid decline of sCr during the first days of life, due to improvement in GFR, may alter the interpretation of the postoperative ΔsCr. Boer et al. [24] have estimated that sCr decreases by 15% with doubling of age before day 65 in healthy neonates. This implies that, beyond day 10, the expected decrease does not exceed about 3% over two days. Taken together with previously published data [25], the 10 day cutoff was chosen to distinguish two groups: patients with an expected physiological decrease in sCr, and others. All analyses were performed separately.

Because of the small mortality rates, analysis employed non parametric methods. First, an exploratory approach was conducted to identify patterns of sCr kinetics in the overall population and association with mortality. Daily ΔsCr was calculated as (sCr-baseline)/baseline x 100 (%) in each patient. All daily ΔsCr within 7 days of surgery were included in individual trajectories of variation, and the Kml algorithm [26] used to identify homogeneous clusters of trajectories. Kml is a partitioning algorithm, an implementation of k-means for longitudinal data, and is used in sociological studies to identify groups of individual developmental trajectories. The aim of the clustering is to minimize the within-cluster distances and maximize the between-cluster distances. The assignment to clusters uses the Euclidean distance. In addition, Kml is able to choose the optimal number of clusters by running k-means several times, by varying the initial number of seeds, and by using the Calinski and Harabasz criterion (a combination of the within- and between -cluster correlation matrices). Because ΔsCr within 2 days of surgery has been shown to be predictive of subsequent AKI in children with CPB [21], the sequence made by ΔsCr on the day of surgery, day 1 and day 2, was used to identify the clusters. Following this, all baseline and intraoperative variables were compared between clusters using the Student t or Mann-Whitney tests, χ^2^ or Fisher tests, as appropriate, and the Bonferroni correction to counteract the problem of multiple comparisons. For mortality, the 95% CIs were estimated by bootstrapping with 2000 re-samples. The cluster including patients with the best short-term outcome was taken as a reference. Additionally, associations between ΔsCr and mortality were assessed by plotting the average mortality rate as a function of ΔsCr. The ability of ΔsCr to discriminate between patients who died within 30-days and survivors was assessed by computing the receiver operating characteristic (ROC) curves and calculating the areas under the ROC curves (AUC). As has been suggested [27], special interest was paid to partial AUCs (pAUC) in the regions of the ROC space where sensitivity is clinically relevant for a biomarker (beyond 0.80). The 95% CIs of all AUCs were estimated by bootstrapping with 2000 re-samples.

Following this, the clinical usefulness of ΔsCr was assessed based on the ability to improve prediction of 30-day mortality. Analysis focused on the daily variation relative to baseline, on pRIFLE [4] and AKIN [6] stages. To focus on early variation, the pRIFLE category was calculated within 2 days of surgery. ΔsCr was added as a variable to a mortality model including clinical variables, and the improvement in discrimination, assessed by the difference in c-indexes and the Integrated Discrimination Improvement (IDI) [28], and the improvement in reclassification, assessed by the category-less Net Reclassification Index (NRI) were calculated [29]. IDI is the integrated difference between sensitivity and 1-specificity, integration being done over all possible cut-offs of the biomarker. As such, it is related to, but more sensitive than the difference in c–indexes. NRI is issued from risk reclassification: any upward reassessment of risk in subjects with the event implies improved reclassification, and any downward reassessment implies worse reclassification. The interpretation is the opposite for subjects without the event. As such, the NRI for events is an indicator of sensitivity of the biomarker, and the NRI for non-events an indicator of specificity [28]. The category-less NRI used here has the advantage of not being affected by event incidence, and allows comparison between studies. Analyses were performed with R software version 2.10.1 for Windows, (www.r-project.org). Clusterization was performed using the “Kml” package in R [26]. Difference in c-indexes, IDI and NRI were calculated using the “Design” package in R.

## Results

Overall 1467 neonates and infants underwent cardiac surgery during the study period, 1104 of whom underwent surgery with CPB. Postoperative sCr concentrations and complete short-term outcome data were available in 1019 patients. They had undergone 36 different procedures, the main procedures are shown in Table 1.

**Table 1:** Surgical procedures performed in the study population.

Closure of venticular septal defect (VSD)	199
Tetralogy of Fallot repair	177
Arterial switch operation (ASO) for transposition of the great arteries	134
Common atrioventricular canal repair	72
ASO and VSD closure	70
Total anomalous pulmonary vein connection repair a	45
Reconstruction of the aortic arch	41
Bidirectional Glenn anastomosis for univentricular heart	31
Aortic stenosis, valvuloplasty	29
Palliation for right ventricular outflow tract obstruction	28
Truncus arteriosus repair	19
ASO, VSD closure and aortic arch repair	19
Modified Blalock-Taussig (systemic to pulmonary) shunt	19
Norwood or Sano procedure for hypoplastic left heart syndrome	17
Anomalous origin of the left coronary artery from the pulmonary artery, repair	16
Other	100

a Of all cases with total anomalous pulmonary vein connection, 20 were restricted, and have undergone emergent surgery, and 25 were unrestricted.

**Figure 1:**
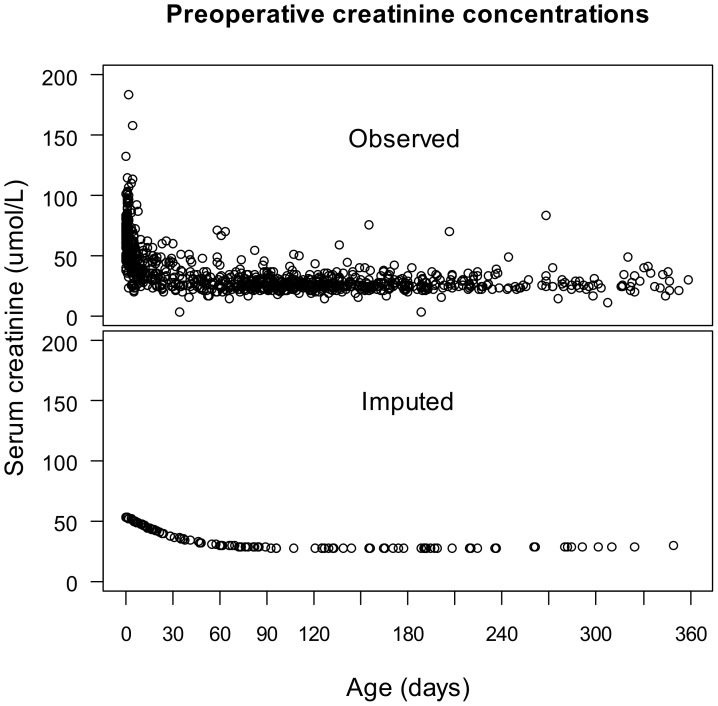
Serum creatinine concentrations prior to surgery as a function of age. The observed concentrations show a trend towards a rapid decrease in creatinine within the first 10 days of life. Missing concentrations and concentrations measured within 2 days of birth were imputed by the expected values from a regression model using age as a predictor.

Preoperative sCr concentrations are plotted in Figure 1 as a function of age. Similar to the findings in healthy infants [24], a rapid decline of sCr was noted during the first 10 days of life, then a more gradual decrease, followed by a plateau beyond 2 months of age. Accordingly, the population was divided into two groups: the patients ≤ 10 days (n = 262), and > 10 days old (n = 757). The preoperative sCr was imputed in 113 patients whose concentrations were missing from their files, and in 172 patients with concentrations measured within 2 days of birth. The median imputed value was 49 μmol L^−1^ (range 27.3 – 53.5 μmol L^−1^). The distributions of the observed and imputed concentrations are shown in Figure 1. Postoperative sCr was available in 960 (94.5%) of all patients on the day of surgery, in 980 (96.4%) on day 1, in 931 (91.6%) on day 2, in 762 (75%) on day 3, in 597 (58.7%) on day 4, in 465 (45.8%) on day 5, in 367 (36.1%) on day 6 and in 297 (26.6%) on day 7 after surgery. Another 23 patients had missing CPB, cross-clamping durations, and ultrafiltration rates, which were imputed as described.

**Figure 2:**
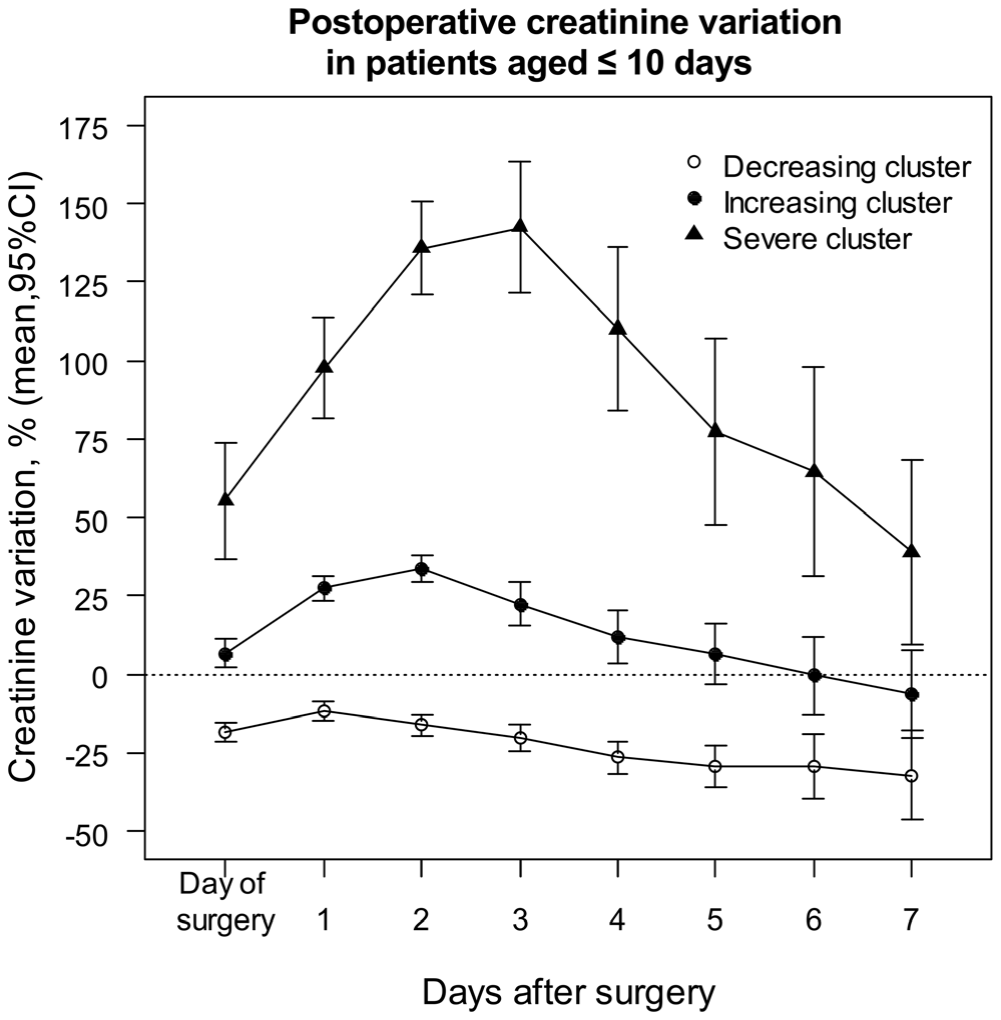
Mean creatinine variation within clusters during the first week of surgery in patients aged ≤ 10 days. Creatinine variation was calculated relative to the baseline concentration. Running the algorithm of clusterization on the individual serum creatinine variation trajectories resulted in three clusters: the “Decreasing cluster” (n = 125), the “Increasing cluster” (n = 101), and the “Severe cluster” (n = 33).

**Figure 3:**
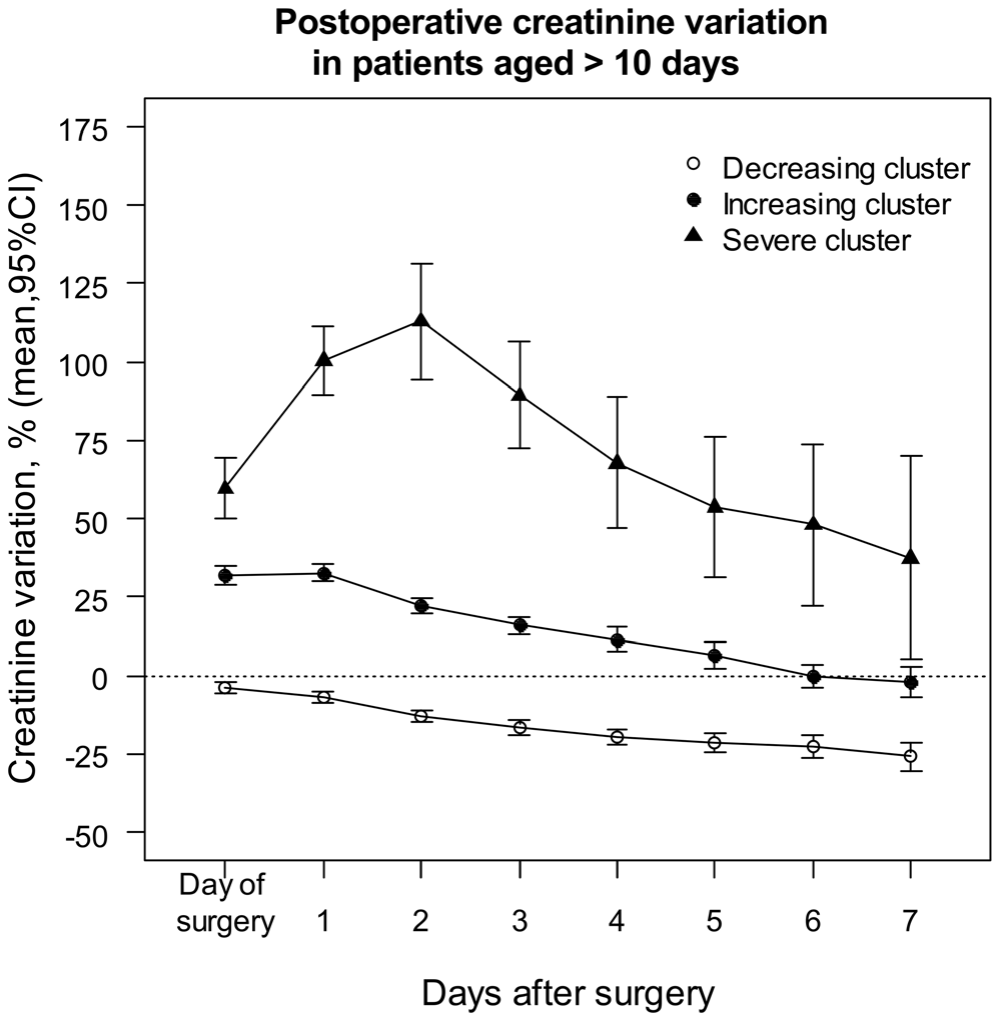
Mean creatinine variation within clusters during the first week of surgery in patients aged > 10 days. Creatinine variation was calculated relative to the baseline concentration. Running the algorithm of clusterization on the individual serum creatinine variation trajectories resulted in three clusters: the “Decreasing cluster” (n = 380), the “Increasing cluster” (n = 317), and the “Severe cluster” (n = 53).

Running Kml on the individual trajectories of ΔsCr resulted in a “Decreasing”, “Increasing”, and a “Severe” cluster in each group (Figures 2 and 3). Seven patients who were discharged from the Intensive Care Unit (ICU) on day 1, and another three who died on day 1 were excluded from clusterization for lack of sufficient sCr measurements to provide a path, but were included in further analysis. Figures 2 and 3 show the average trajectories per cluster within the first week of surgery. Although the clusterization relied exclusively on the first three values of each path, there was no subsequent overlap of the 95% confidence intervals (CI), suggesting that ΔsCr within 48h was predictive of the entire path. In both groups, the “Decreasing” cluster included patients with short CPB and cross-clamping durations, with few blood product transfusions, and a the majority of short and uncomplicated recoveries (Tables 2 and 3), and was taken as a reference. The mean sCr decreased by about 25% during the first week in patients aged ≤ 10 days, and by about 5 – 15% in older ones. In both populations, patients in the “Increasing” clusters had longer CPB durations and larger transfusions, required peritoneal dialysis more often, were discharged one day later and had a non-significantly higher mortality rate. Their sCr increased by about 25% until day 2, and decreased towards the baseline within one week. In both groups, patients in the “Severe” cluster had undergone the longest and most complex procedures, and had large transfusions. They had a longer ICU stay, more than a half had AKI requiring dialysis, and they had a significantly higher mortality rate. Their sCr increased by more than 50% for several days.

**Table 2:** Patient baseline, intraoperative and postoperative characteristics within the clusters in patients aged ≤ 10 days.

Variable		“Decreasing	“Increasing	P-value a	“Severe	P-value a
		cluster”	cluster”		cluster”	
		(n = 125)	(n = 101)		(n = 33)	
Age (days)		5.7±2.0	5.7±2.4	0.88	5.0±3.0	0.23
Weight (Kg)		3.3±0.5	3.1±0.5	0.06	3.2±0.4	0.27
Aristotle score b		10.1±5.1	10.0±1.6	0.81	10.7±2.2	0.32
Re-sternotomy, n (%)		3 (2.4)	4 (4.0)	0.70	0	0.99
Baseline serum creatinine (mmol L^−1^)^c^		60.7±24.3	44.0±9.7	<0.001	30.3±8.5	<0.001
Duration of cardiopulmonary bypass (min)		114.3±36.0	133.9±53.2	0.002	169.5±57.8	<0.001
Duration of aortic cross-clamping (min)		67.8±28.7	71.9±28.7	0.28	79.3±3.8	0.09
Surgery with deep hypothermic circulatory arrest		7 (5.6)	15 (14.8)	0.02	20 (60.6)	<0.001
Priming volume (mL)		363.8±72.4	357.1±75.9	0.50	343.5±81.4	0.20
Ultrafiltration rate (mL min^−1^bypass per kg body weight)		2.0±0.9	1.9±0.6	0.37	1.8±0.9	0.50
Ultrafiltration rate (mL min^−1^bypass per m^2^BSA)		51.0±23.4	48.1±16.0	0.26	47.4±22.4	0.42
Received aprotinin		49 (39.2)	52 (51.5)	0.06	12 (36.4)	0.77
Number of blood products on day of surgery, median, IQR		4, 3 – 5	4, 4 – 5	0.03	4, 4 – 6.2	<0.001
Required delayed sternal closure		16 (12.8)	29 (28.7)	0.003	23 (69.7)	<0.001
Required postoperative ECMO		2 (1.6)	2 (2.0)	0.99	2 (6.1)	0.19
AKI stage according to pRIFLE	“Risk”	1 (0.8)	57 (56.4)	<0.001	5 (15.2)	<0.001
	“Injury”	0	0		28 (84.8)	
	“Failure”	0	0		0	
AKI stage according to AKIN	stage 1	0	21 (20.8)	<0.001	2 (60.6)	<0.001
	stage 2	0	0		5 (15.1)	
	stage 3	2 (1.6)	16 (15.8)		26 (78.8)	
Required postoperative peritoneal dialysis		2 (1.6)	16 (15.8)	<0.001	25 (75.7)	<0.001
Delay to peritoneal dialysis (days), median, IQR		2.5, 1.2 – 3.7	0, 0 – 1	0.54	0, 0 – 1	0.37
Required re-operation ≤ 48 hours of surgery		1 (0.8)	4 (4.0)	0.17	0	0.99
Duration of mechanical ventilation (days), median, IQR^d^		1, 1 – 3	2, 1 – 5	0.01	5, 4 – 7.5	<0.001
Length of Intensive Care Unit stay (days), median, IQR^d^		4, 3 – 6	5, 3.5 – 8.5	0.03	9, 6 – 13	<0.001
30-day mortality		2 (1.6)	6 (5.9)	0.14	10 (30.3)	<0.001

Data are shown as means and standard deviations, or as numbers and percentages, unless stated otherwise.

a the “Decreasing cluster” was taken as reference. According to the Bonferroni correction, the significance level was set to 0.025.

b an expert-based stratification of the surgical complexity, potential for morbidity and mortality, ranging between 1.5 and 15 [22].

c only reliable measurements were compared.

d in 30-day survivors.

ECMO: extracorporeal membrane oxygenation.

**Table 3:** Patient baseline, intraoperative and postoperative characteristics within the clusters in patients aged > 10 days.

Variable		“Decreasing	“Increasing	P-value a	“Severe	P-value a
		cluster”	cluster”		cluster”	
		(n = 380)	(n = 317)		(n = 53)	
Age (days)		136.9 84.4	126.9±79.5	0.11	102.4±78.7	0.004
Weight (Kg)		5.0±1.5	4.9±1.5	0.35	4.4±1.5	0.003
Aristotle score b		7.8±1.7	8.1±1.8	0.01	9.0±1.5	<0.001
Re-sternotomy, n (%)		74 (19.5)	41 (12.9)	0.02	6 (11.3)	0.15
Baseline serum creatinine (mmol L^−1^)^c^		31.6±10.1	27.6±8.0	<0.001	31.4±10.7	0.90
Duration of cardiopulmonary bypass (min)		98.6±54.6	115.7±78.5	0.001	170.7±81.0	<0.001
Duration of aortic cross-clamping (min)		51.0±26.3	62.5±33.9	<0.001	86.0±35.5	<0.001
Surgery with deep hypothermic circulatory arrest		13 (3.4)	17 (5.4)	0.21	3 (5.7)	0.43
Priming volume (mL)		381.5±71.4	390.4±72.6	0.11	383.1±37.3	0.80
Ultrafiltration rate (mL min^−1^bypass per kg body weight)		1.4±0.8	1.5±0.8	0.69	1.5±0.6	0.95
Ultrafiltration rate (mL min^−1^bypass per m^2^BSA)		42.3±20.2	42.6±19.8	0.80	40.3±14.5	0.39
Received aprotinin		134 (35.3)	128 (40.4)	0.06	25 (47.2)	0.05
Number of blood products on day of surgery, median, IQR		3, 2 – 4	4, 3 – 4	0.009	5, 4 – 6.2	<0.001
Required delayed sternal closure		16 (4.2)	47 (14.8)	<0.001	25 (47.2)	<0.001
Required postoperative ECMO		1 (0.3)	4 (1.3)	0.18	3 (5.7)	0.006
AKI stage according to pRIFLE	“Risk”	15 (3.9)	208 (65.6)	<0.001	20 (37.7)	<0.001
	“Injury”	0	10 (3.1)		31 (58.5)	
	“Failure”	0	0		2 (3.8)	
AKI stage according to AKIN	stage 1	2 (0.5)	102 (32.2)	<0.001	16 (30.2)	<0.001
	stage 2	0	8 (2.5)		13 (24.5)	
	stage 3	8 (2.1)	17 (5.3)		24 (45.3)	
Required postoperative peritoneal dialysis		8 (2.1)	17 (5.4)	0.02	21 (39.6)	<0.001
Delay to peritoneal dialysis (days), median, IQR		0.5, 0 – 1	0, 0 – 1	0.99	0, 0 – 1	0.38
Required re-operation ≤ 48 hours of surgery		2 (0.5)	4 (1.3)	0.42	0	0.99
Duration of mechanical ventilation (days), median, IQR^d^		0, 0 – 3	1, 0 – 5	0.006	8, 4 – 13	<0.001
Length of Intensive Care Unit stay (days), median, IQR^d^		3, 2 – 6	4, 2 – 8	0.003	12, 6 – 22	<0.001
30-day mortality		6 (1.6)	12 (3.8)	0.07	8 (15.1)	<0.001

Data are shown as means and standard deviations, or as numbers and percentages, unless stated otherwise.

a the “Decreasing cluster” was taken as reference. According to the Bonferroni correction, the significance level was set to 0.025.

b an expert-based stratification of the surgical complexity, potential for morbidity and mortality, ranging between 1.5 and 15 [22].

c only reliable measurements were compared.

d in 30-day survivors.

ECMO: extracorporeal membrane oxygenation.

Overall, 47 patients (4.6%) died within 30 days of surgery. Mortality rates and the estimated 95% CIs were: 1.6% [0.0 – 4.1] in the “Decreasing”; 5.9% [1.9 – 10.9] in the “Increasing”; and 30.3% [15.1 – 46.2] in the “Severe” cluster in patients ≤10 days old, and were 1.6% [0.5 – 3.0], 3.8% [1.9 – 6.00] and 15.1% [5.9 – 25.5], respectively, in older ones. As shown in Tables 2 and 3, and by the overlap of the 95% CIs, mortality rates differed significantly between the “Decreasing” and “Severe” clusters, but not between the “Decreasing” and the “Increasing” clusters. Of all the patients who died, 18.2% were in the “Decreasing”, 40.9% were in the “Increasing” and 40.9% were in the “Severe” clusters. Overall, 89 patients required peritoneal dialysis, 11.2% were in the “Decreasing”, 37.1% were in the “Increasing”, and 51.7% were in the “Severe” clusters. Of all the patients who required dialysis 27 died (30.3%), compared to 20 out of 930 patients without dialysis (2.1%), p<0.001.

**Figure 4:**
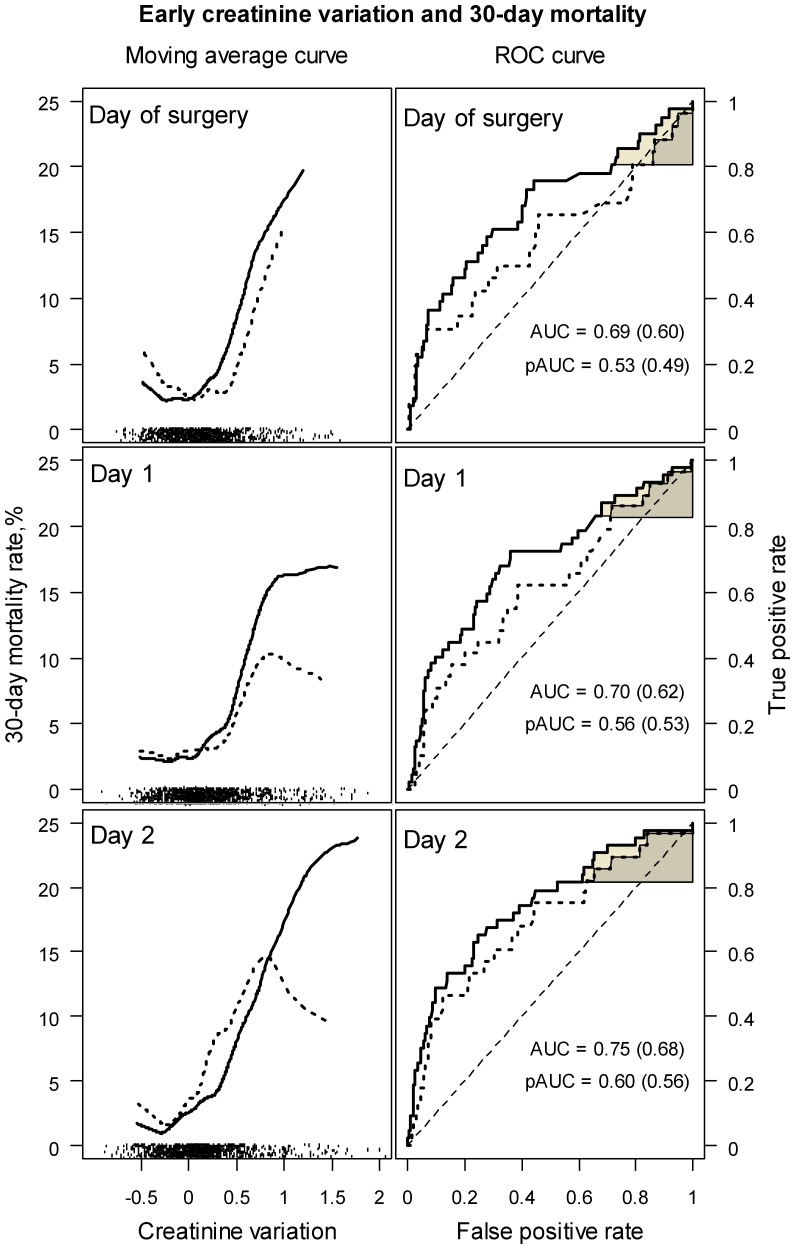
30-day mortality as a function of creatinine variation within 2 days of surgery (on the left), and ROC curves for the prediction of 30-day mortality by creatinine variation within 2 days of surgery (on the right). Dotted lines were drawn after exclusion of cases with imputed preoperative creatinine concentrations. The U shaped curves on the left show that the nadir of the mortality rate was reached for a decrease in creatinine of about -25%, which may represent the normal course. Data density is shown as tick marks on the bottom of each portion of the figure. Creatinine variation is not informative of the risk of death between –50% and +50%, which covers the highest data density. On the right, the overall and partial ROC areas (AUC and pAUC) show poor prediction of 30-day mortality by the postoperative creatinine variation. ROC areas in brackets were calculated after exclusion of cases with imputed preoperative creatinine concentrations.

Figure 4 shows, on the left, the average mortality rate as a function of ΔsCr within 2 days of surgery. Mortality was high in patients with a > 50% increased sCr. However, the mortality rate observed in patients with a 50% decrease was close to that observed in patients with a 50% increase in sCr, and resulted in a U-shaped curve. This region of the curve covered the highest data density (shown on the bottom of the figure), and suggests a weak relationship between ΔsCr and mortality in a majority of patients. Eight patients died in the “Decreasing” clusters, their sCr decreased by median –14.1% on day 1 (range –56.1% to –2.7%), and by median –9.7% on day 2 (ranged –65.8% to –3.5%). Only one of them had dialysis. The receiver operating curves (ROC) plotted in Figure 4, on the right, suggest poor discrimination between survivors and patients who died by the ΔsCr, with areas under the curve (AUC) of 0.687, 95% CI [0.593 – 0.778] on the day of surgery, 0.704 [0.610 – 0.784] on day 1 and 0.746 [0.663 – 0.825] on day 2. Discrimination was even worse when patients with missing preoperative sCr were excluded, as shown by the dotted curves and by the AUCs of 0.598 [0.472 – 0.729], 0.620 [0.495 – 0.740] and 0.680 [0.564 – 0.794], respectively. Partial areas under the ROC curve (pAUC) for a sensitivity bound of 0.80 demonstrated near random classifications: 0.532 [0.485 – 0.645] on the day of surgery, 0.561 [0.501 – 0.648] on day 1, and 0.601 [0.532 – 0.711] on day 2, and 0.492 [0.458 – 0.571], 0.535 [0.475 – 0.613], and 0.564 [0.491 – 0.678], respectively, after the exclusion of patients with missing preoperative sCr.

The variables tested for the 30-day mortality model were: age, weight, the Aristotle score, the durations of CPB and cross-clamping, the use of DHCA and the requirement for postoperative extracorporeal membrane oxygenation. The model included the duration of CPB (regression coefficient 0.01±0.004, p <0.001) and the use of DHCA (regression coefficient 1.67±0.54, p = 0.02) in patients ≤ 10 days old, and had good calibration (p = 0.89) and discrimination (c-index  = 0.861). In older patients, the model included the duration of CPB (regression coefficient 0.004±0.001, p = 0.02) and the Aristotle score (regression coefficient 0.32±0.12, p = 0.006), and had acceptable calibration (p = 0.12) and discrimination (c-index = 0.713). Early ΔsCr was added to the mortality model either as the daily variation, or as the pRIFLE [4] or AKIN stage [6]. As shown in Table 4, either daily ΔsCr or pRIFLE increased the c-index substantially over the c-index of the initial model. Changes in IDI were not significant either. Adding daily ΔsCr or pRIFLE resulted in reclassification of the risk of death, but the predictions in patients who died (NRI for events) did not improve, suggesting low sensitivity of ΔsCr. In contrast, the AKIN classification, which additionally includes dialysis requirement for AKI staging [6], significantly improved discrimination in both groups, and improved NRI in patients aged ≤ 10 days who died. On the other hand, ΔsCr improved predictions in patients who survived (NRI for non events), implying excellent specificity.

**Table 4:** Improvement in discrimination and in reclassification of the risk of death within 30-days.

Timing of measurement	Day 0	Day 1	Day 2	pRIFLE a	AKIN
		Patients aged	≤ 10 days		
Difference in c-indexes	0.053	0.034	0.026	0.036	0.059
with 95% CI	[–0.084 – 0.215]	[–0.103 – 0.199]	[–0.105 – 0.189]	[–0.114 – 0.189]	[–0.085 – 0.196]
	p = 0.19	p = 0.41	p = 0.53	p = 0.38	p = 0.15
IDI with 95% CI	0.087	0.010	0.024	0.016	0.066
	[0.022 – 0.151]	[–0.018 – 0.038]	[–0.020 – 0.068]	[–0.027 – 0.059]	[0.019 – 0.113]
	p = 0.09	p = 0.47	p = 0.28	p = 0.48	p = 0.006
NRI for events	0.238	0.048	0.000	0.048	0.524
with 95% CI	[–0.190 – 0.666]	[–0.380 – 0.475]	[–0.462 – 0.462]	[–0.380 – 0.475]	[0.096 – 0.952]
	p = 0.27	p = 0.83	p = 1	p = 0.83	p = 0.02
NRI for non-events	0.543	0.504	0.527	0.463	0.602
with 95% CI	[0.414 – 0.672]	[0.379 – 0.629]	[0.401 – 0.653]	[0.338 – 0.588]	[0.477 – 0.727]
	p<0.001	p<0.001	p<0.001	p<0.001	p<0.01
		Patients aged	> 10 days		
Difference in c-indexes	0.012	0.016	0.016	0.038	0.064
with 95% CI	[–0.183 – 0.185]	[–0.162 – 0.199]	[–0.128 – 0.205]	[–0.114 – 0.185]	[–0.069 – 0.222]
	p = 0.78	p = 0.69	p = 0.69	p = 0.35	p = 0.12
IDI with 95% CI	0.016	0.005	0.021	0.024	0.043
	[–0.004 – 0.037]	[–0.009 – 0.020]	[–0.018 – 0.061]	[–0.004 – 0.051]	[0.016 – 0.710]
	p = 0.12	p = 0.47	p = 0.28	p = 0.09	p = 0.002
NRI for events	0.077	0.000	0.120	0.077	0.231
with 95% CI	[–30.7 – 46.1]	[–38.4 – 38.4]	[–0.272 – 0.512]	[–0.307 – 0.461]	[–0.154 - 0.615]
	p = 0.69	p = 1	p = 0.55	p = 0.69	p = 0.24
NRI for non-events	0.101	0.303	0.292	0.267	0.576
with 95% CI	[0.027 – 0.176]	[0.230 – 0.376]	[0.215 – 0.369]	[0.194 – 0.340]	[0.503 – 0.648]
	p = 0.03	p<0.001	p<0.001	p<0.001	p<0.001

After creatinine variation was added to a 30-day mortality prediction model, improvement in discrimination was assessed by the difference in c-indexes and by the Integrated Discrimination Improvement [27], and improvement in reclassification by the category-less Net Reclassification Index [28]. The 30-day mortality model included the length of bypass and requirement for deep hypothermic circulatory arrest in patients under 10 days of age, and included the length of bypass and the Aristotle score [22] in older ones.

a for the purpose of this study, the pRIFLE stage was assessed within 2 days of surgery.

IDI: Integrated Discrimination Improvement; NRI: Net Reclassification Index.

## Discussion

The present findings suggest that a postoperative decrease in sCr represents the normal course in neonates and infants undergoing cardiac surgery with CPB. The assessment of AKI severity by the risk of postoperative death has highlighted the lack of sensitivity of early changes in sCr for the detection of kidney injury.

Three clusters of postoperative sCr kinetics were identified in the present population. SCr decreased in 50% of all patients, a profile which has not been reported in infants with CPB to date, and was associated with the best outcome. Another 41.4% had an average 25% transient increase in sCr, increased morbidity but, unlike in adults with cardiac surgery [10], no significant increase in 30-day mortality. When ΔsCr varied between –50% and +50%, which covered the majority of the cases here, an U-shaped relationship was observed between the average mortality rate and ΔsCr, suggesting that ΔsCr is not informative of the risk of death in a the majority of the present population. Only an abrupt ≥ 50% increase in postoperative sCr, found in 8.6% of all patients, was associated with an increase in the risk of death. This is in accordance with previous literature, showing an abrupt increase in sCr be associated with a worse outcome in infants with cardiac surgery. In young infants Blinder et al. [17] reported a 100–200% increase in sCr in 14%, and a >200% increase in 7%, both of which were statistically associated with the risk of in-hospital death, longer durations of ventilation, inotropic support and ICU stay [17]. In neonates, Morgan et al. reported significantly higher mortality rates when sCr doubled or beyond, and longer durations of ventilation and ICU-stay [19]. However, an increase in sCr of > 50% occurred only in one half of all patients who required dialysis or died in the present cohort, suggesting low sensitivity of early ΔsCr for the diagnosis of severe AKI.

Following a recent recommendation [16], in the present analysis, AKI severity was accounted for by the risk of postoperative death. Accordingly, if early ΔsCr was a reliable marker of the changes in kidney function, then early ΔsCr would reliably predict the risk of death. However, early ΔsCr was a poor classifier between survivors and patients who died, and did not improve predictions in patients who died, nor discrimination, compared to a model including clinical predictors. These imply whether that the kidney damage is not associated with the risk of death in the present setting, or that ΔsCr is not an early and/or reliable marker of the kidney damage. It is unlikely that survival is unaffected by changes in kidney function, a common finding in adults undergoing cardiac surgery [7]–[10]. Here, the prediction of death was significantly improved when AKI was additionally assessed by the dialysis requirement. The slow sCr kinetics [3] cannot entirely explain the lack of predictive ability either, as early ΔsCr has previously been shown to predict the subsequent sCr kinetics [21], and was indicative of the entire path here (Figures 2 and 3). The risk of death was not associated with ΔsCr taken over the entire range of variation, but large increases in sCr were associated with higher mortality rates (in the “Severe” clusters). Finally, ΔsCr improved predictions in survivors. All together, these are reasons to believe that sCr has good specificity but lacks sensitivity for the assessment of cardiac surgery related AKI in patients aged < 1year.

A new finding in the present study is the “Decreasing” cluster, including patients with short CPB durations and few blood product transfusions, with a low mortality rate, and a short ICU stay. Ho et al. reported had a >10% decrease of in sCr postoperatively in 52% of adults undergoing surgery with CPB [11], and Lassnigg et al reported a decrease in sCr in 54% of a cohort of 3123 adults with cardiac and thoracic aortic surgery. As reported by Lassnigg et al [8], [9] the mortality rate reached a nadir in patients in whom sCr decreased by 0.3 mg/dL, increased in patients in whom sCr remained unchanged or increased by up to 0.5 mg/dL, and was high in patients in whom sCr increased by ≥ 0.5 mg/dL. The reason why a slight sCr decrease was protective could not be explained based on the reported data, and it was assumed that the decrease in sCr must have been the result of a dilutional effect during CPB in patients with normal GFR, and might represent the normal course in adults. Importantly, large mortality rates were observed in patients in whom sCr decreased by more than 0.3mg/dl, resulting in a U shaped curve similar to that shown in Figure 4. In the present study the risk of death reached a nadir in patients with an about 25% decrease in sCr, and increased in patients in whom sCr dropped. However, the small sample size in this study did not allow establish a cut-off for the prediction of 30-day mortality in patients with decreased sCr.

It is conceivable that patients aged ≤ 10 days old have a decreased sCr postoperatively, due to a physiological increase in GFR. In patients > 10 days old, however, the physiological decrease in sCr could not exceed 3% over two days, and cannot explain the “Decreasing “cluster. Several other factors may explain the early decrease in sCr here. The first is massive hemodilution, due to the disproportion between the patient’s size and the extracorporeal volume, and is responsible for of a 50% reduction in coagulation factors, a 70% drop in platelet count [30], and probably of a similar decrease in sCr. The second is ultrafiltration, which has become common practice to limit transfusions and inflammation associated with CPB [31]. In the present study, the ultrafiltration rate was set at 1.5–2 ml min^−1^ CPB per body weight: this rate equated to 40 –50 ml min^−1^ CPB per m^2^ body surface. Normal GFR is 23.7±8.7 ml min^−1^ m^−2^ in neonates less than 1 week of age, and improves to 57.2±12.7 ml min^−1^ m^−2^ in infants [32]. Accordingly, sCr by the end of bypass may simply indicate the point at which creatinine clearance, whether performed by the glomerulus or by the bypass filter, comes into balance with production, and it is conceivable that compensation of an impaired GFR by intra-operative ultrafiltration might have occurred in very young patients but not in older ones. Altogether, by the end of bypass, hemodilution, ultrafiltration and GFR are expected to result in decreased sCr in patients with normal GFR, and an increase in sCr may imply a severely impaired GFR.

The dialysis and mortality rates among the patients in the “Decreasing” clusters suggest another phenomenon: fluid overload. The distribution volume of creatinine is equivalent to total body water. The dilutional effect of fluid overload results in delayed recognition and/or underestimation of AKI severity in adults [33], [34]. The degree of fluid overload at the initiation of RRT is a risk factor of death in adults and children [35]–[39], and lower sCr concentrations at the initiation of RRT are associated with a higher risk of death [8], [9], [40], [41]. The U-shaped curve in Figure 4 suggests an increase in mortality when the negative ΔsCr was large, which might have been due to fluid overload. Unfortunately, no objective means to assess fluid overload was retrospectively available here.

### Limitations

The retrospective design of the present study requires that the validity of our findings be considered with caution. Preoperative sCr was imputed in 28% of all patients, using a linear regression methodology which has not been validated to date. Bilirubin concentrations rise after birth, and may rise by the end of CPB due to hemolysis, High bilirubin concentrations may have a tremendous impact on the interpretation of sCr with the Jaffé technique [42]; however, bilirubin concentrations were not analyzed. Fluid overload, which alters sCr concentrations, was not assessed either. Due to the high prevalence of missing data beyond day 2, the analysis was restricted to 48 h following surgery. The initiation of dialysis may have resulted in a biased estimation of the postoperative ΔsCr in several patients.

## Conclusions

The present results suggest that a postoperative decrease in serum creatinine represents the normal course in neonates and infants with cardiac surgery. Knowing the extent to which creatinine changes postoperatively adds little information for the prediction of the risk of death, and only an abrupt increase, occurring in a minority of patients, is associated with postoperative mortality. Also, the present findings suggest that early creatinine variations lack sensitivity for the assessment of the severity of kidney injury in neonates and infants with cardiac surgery.
